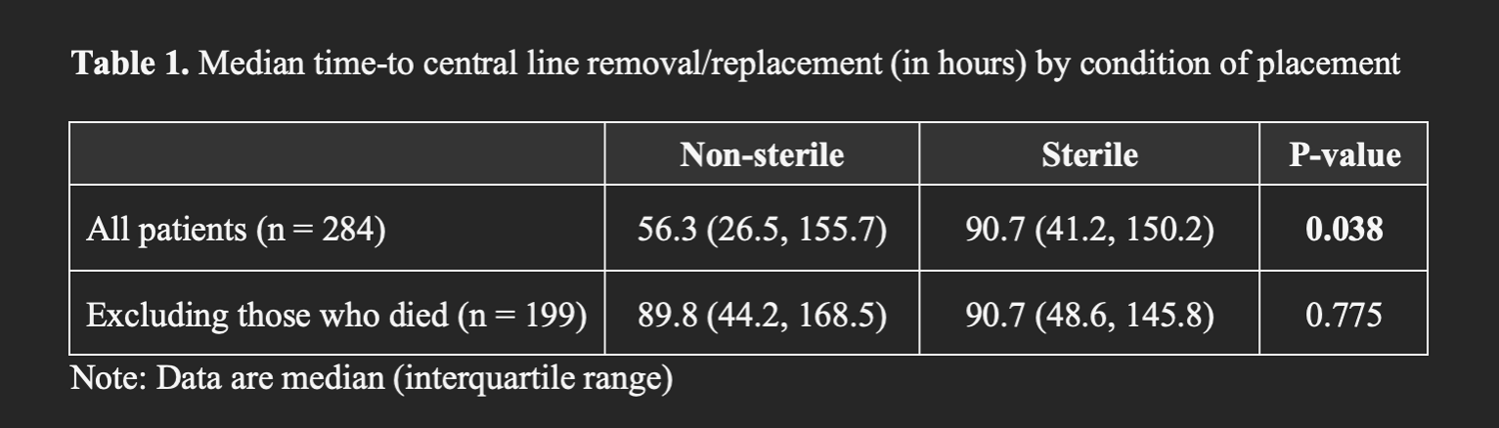# 162 The SSI Standstill: Are Stagnant SSI Rates Limited to CMS-Mandated Procedures?

**DOI:** 10.1017/ash.2026.10563

**Published:** 2026-06-23

**Authors:** Frank Frankovsky, Jennifer Clary, Stephanie Stroever, Thane Thomas, Joshua Preator, James Morris, Mark Sigler

**Affiliations:** 1 Texas Tech University Health Science Center; 2 UMC Healthsystem; 3 Texas Tech University Health Sciences Center; 4 TTUHSC

## Abstract

**Authors and Affiliations:** Frank Frankovsky III, Thane Thomas, Mark Sigler, MD, Joshua Preator, DO, James Morris, MD, MPH, FACEP, Jennifer Clary, MSN, RN, CIC, Stephanie Stroever, PhD, MPH **Background:** Healthcare Infection Control Practices Advisory Committee (HICPAC) guideline 2.2.11 recommends replacement of central venous catheters inserted emergently under non-sterile conditions as soon as possible and ideally within 48 hours. The extent of compliance with this recommendation and associated outcomes in emergency department (ED)–placed central lines remains unstudied. Methods We conducted an observational study of adult patients admitted following central line placement in the ED at two hospitals in West Texas between June 2024 and August 2025. Patients who died in the ED were excluded. The primary outcome was time to central line removal or replacement after admission. Central lines were classified as emergent (non-sterile) if placed without maximal sterile barrier precautions and documented as such in a standardized ED log. Conditions of line placement were verified by investigator chart review and, when necessary, direct discussion with the team involved in placement. Patient demographics, indications for line placement, and clinical outcomes were abstracted from the medical record. Median time to removal or replacement was calculated for sterile and non-sterile lines and compared using the Wilcoxon rank-sum test; p-values <0.05 were considered statistically significant. Sensitivity analyses excluded patients who died after hospital admission. Results A total of 284 patients were included, of whom 121 (42.6%) had non-sterile central lines placed. Mean age was 64.5 years, with similar distribution by sex. Common indications for line placement included altered mental status (24.3%), cardiac arrest (19.0%), and shortness of breath (14.8%). Median time to removal/replacement was shorter for non-sterile lines than sterile lines (56.2 vs 90.7 hours; p = 0.038). This difference was not observed in sensitivity analyses excluding patients who died after admission (p = 0.775), suggesting survivorship or competing risk bias. No central line–associated bloodstream infections (CLABSI) were identified. Conclusion No CLABSI events were observed despite median central line dwell times exceeding the 48-hour threshold referenced in HICPAC guidance. Early mortality among patients with emergently placed lines may have limited the opportunity for infection to occur. These findings emphasize the role of concurrent infection prevention measures and highlight the need for multidisciplinary strategies to support timely replacement of emergently placed central lines.

**Figure:**